# Identification and Characterization of New *Hafnia* Strains from Common Carp (*Cyprinus carpio*), Potentially Possessing Probiotic Properties and Plastic Biodegradation Capabilities

**DOI:** 10.3390/ijms26031119

**Published:** 2025-01-28

**Authors:** Luka Dragacevic, Darya Tsibulskaya, Milan Kojic, Nevenka Rajic, Aleksandar Niksic, Mina Popovic

**Affiliations:** 1 Institute of Virology, Vaccines and Sera, Torlak, Vojvode Stepe 458, 11221 Belgrade, Serbia; ldragacevic@torlak.rs (L.D.); 2650460@gmail.com (D.T.); mkojic@torlak.rs (M.K.); 2 Faculty of Ecology and Environmental Protection, University Union-Nikola Tesla, Cara Dušana 62-64, 11158 Belgrade, Serbia; nrajic@unionnikolatesla.edu.rs; 3 Faculty of Veterinary Medicine, Department of Microbiology, University of Belgrade, Bulevar oslobođenja 18, 11000 Belgrade, Serbia; aleksandar.niksic@vet.bg.ac.rs

**Keywords:** probiotics, *Hafnia alvei*, *Hafnia paralvei*, WGS, plastic biodegradation

## Abstract

Finding and characterizing new bacterial strains, including probiotic strains, is a crucial task in today’s world to expand the genetic data pool and identify new genes. In this study, we investigated the gut microbiota of one industrial species, *Cyprinus carpio*, and identified representatives of various microbial genera, including *Citrobacter*, *Serratia*, *Bacillus*, *Enterococcus*, and *Kocuria*. Notably, we discovered two strains of *Hafnia* with potentially probiotic properties. We conducted next-generation sequencing (NGS) of these strains, described their antibiotic resistance and antibacterial activity, and compared them with other representatives of the *Hafnia* genus. These strains, characterized by rapid growth, the presence of the *ClpB* heat shock protein gene, and genes associated with microplastic degradation, provide a promising basis for further research, including studies on their potential application in plastic biodegradation.

## 1. Introduction

The common carp (*Cyprinus carpio*) is a significant contributor to the global aquaculture industry, with a yearly output of 4.1 million tons, accounting for 7.7% of the total freshwater aquaculture production [1]. *C. carpio* is found in freshwater globally, including the rivers and lakes of Serbia. Its global importance is due to its nutritious composition, exceptional flesh quality, capacity to flourish in cold climates, and adequate food conversion.

The gastrointestinal tract (GIT) serves as the principal reservoir of bacteria, viruses, and fungi in fish, while its composition is affected by environmental contamination in aquatic ecosystems. The gut microbiota is a complex system influenced by various factors, including nutrition, changes in the water environment, and the health status of the fish [2]. It is vital for the digestion of indigestible nutrients, detoxification, synthesizing essential nutrients and vitamins, regulating immune homeostasis, and maintaining health. *Proteobacteria*, *Bacteroidetes*, *Firmicutes*, *Fusobacteria*, and *Spirochaetes* are the most prevalent families of bacteria found in common carps [3]. GIT is a valuable reservoir for the discovery of new species of bacteria; an orange to pink-colored bacterial strain that was recently isolated from the GIT of mirror carp, *C. carpio* var. *specularis* (*Lacepède*, 1803), designed as CT19T, is coccoidal, aerobic, Gram-positive, non-motile, and non-spore-forming bacterium. The strain was found to have a close relationship with *Salinicoccus hispanicus* J-82T, according to the sequence of the 16S rRNA gene [4]. Moreover, another study conducted in vitro showed that the strains of *Levilactobacillus brevis* isolated from the intestines of *C. carpio* exhibit promising probiotic properties. The strains exhibit characteristics typical for probiotic bacteria and have no unfavorable features [5]. In the study conducted by Li and coworkers (2023), the intestine of a healthy common carp (*Cyprinus carpio*) was used to isolate a novel strain of *Enterobacter asburiae* designed as E7. This strain exhibited a broad antibacterial spectrum against a variety of bacteria, including *Aeromonas hydrophila*, *A. veronii*, *A. caviae*, *A. media*, *A. jandaei*, *A. enteropelogenes*, *A. schubertii*, *A. salmonicida*, *Pseudomonas aeruginosa*, *Ps. putida*, *Plesiomonas shigelloides*, and *Shewanella* [6]. The results show that strain E7 was not harmful to the host and was sensitive to most antibiotics used in human clinical practice.

Moreover, studying the microbiota of *C. carpio* may be valuable in addressing another global pressing issue—plastic biodegradation. Modern aquatic ecosystems are accumulating significant amounts of microplastic, creating conditions that favor the proliferation of microorganisms capable of degrading this material [7]. Thus, the intestinal tract *of C. carpio* serves as a potential reservoir for discovering such species. One potentially beneficial microorganism could be representatives of the *Hafnia* genus, such as *Hafnia alvei*.

*H. alvei* is a rod-shaped, Gram-negative, facultatively anaerobic bacterium representing a rare isolated species within a new generation of probiotics [8]. This bacterium is one of numerous *Enterobacteriaceae* species of the phylum *Proteobacteria*, found in the GIT of *C. carpio*. It can also be extracted from the GIT of other animals, plant surfaces, soil, and water, as well as from medical waste and materials [9]. *H. alvei* remain insufficiently studied; it may function as a commensal organism inside the GIT. However, it also has the potential to act as an opportunistic pathogen, contributing to numerous diseases [8,10]. It is widely considered a commensal bacterium, signifying it coexists with its host without causing harm. It is proposed that it has a role in regulating gut microbiota and may benefit the host’s health. It is essential to notice that *H. alvei* can cause sickness in particular circumstances, such as in patients with compromised immune systems or those receiving chemotherapy [11]. The bacteria may elicit symptoms such as diarrhea, abdominal pain, and fever in these cases. Still, it is significant for its potential as a probiotic to maintain a balanced gut microbiota. The subspecies *Hafnia paralvei* is classified as a Gram-negative foodborne pathogenic bacterium in several aquatic organisms and seafood. The pathogenesis of these bacteria relies on biofilm formation/development as the primary virulence factor. Consequently, the pathogenicity of these strains depends not only on the mechanisms involved in the biofilm formation but also on the dispersion of the biofilm, which affects the swift/rapid proliferation of these strains [12].

In recent years, substantial research has been conducted on the antimicrobial resistance and drug susceptibility of *H. alvei* [13,14,15]. The published results demonstrate that the *H. alvei* strains are sensitive to many drugs. Nonetheless, scant information exists regarding the tests performed under clinical and epidemiological laboratory conditions and the existing data on the possible pathogenicity of the *H. alvei* strain [14]. The *H. alvei* strain has been extensively studied for its antimicrobial resistance and antibiotic susceptibility. It is sensitive to various antibiotics, but there is limited knowledge about its potential pathogenicity [16,17,18]. Recent studies in food and medical sciences have shown its preventive role as a probiotic, protecting against harmful microorganisms and weakening pathogen virulence. The commercially available probiotic protective culture, LALCULT^®^ Protect *H. alvei* B16, restricts *Escherichia coli* proliferation in cheese and *Salmonella* growth in food [18,19].

In this study, novel strains of *H. alvei* and *H. paralvei* were discovered and isolated from *C. carpio*, and the goal of this work was to provide a detailed description/characterization of these strains. Since these strains can act as probiotics and combat various pathogens harmful to the host, a thorough characterization of isolated strains was done. As part of the screening process for the strains, the method of sequencing the 16S rRNA gene was used. The strains *H. alvei* UUNT_MP41 and *H. paralvei* UUNT_MP29 were thoroughly investigated using whole genome sequencing (WGS) to identify the traits that set them apart precisely. In addition, research was carried out to explore the antibacterial sensitivity of probiotic culture, its antimicrobial activity, and other vital characteristics.

## 2. Results

### 2.1. Identification of Autochthonous Strains Using 16S rRNA Sequencing, Isolated from C. carpio

**Figure 1:**
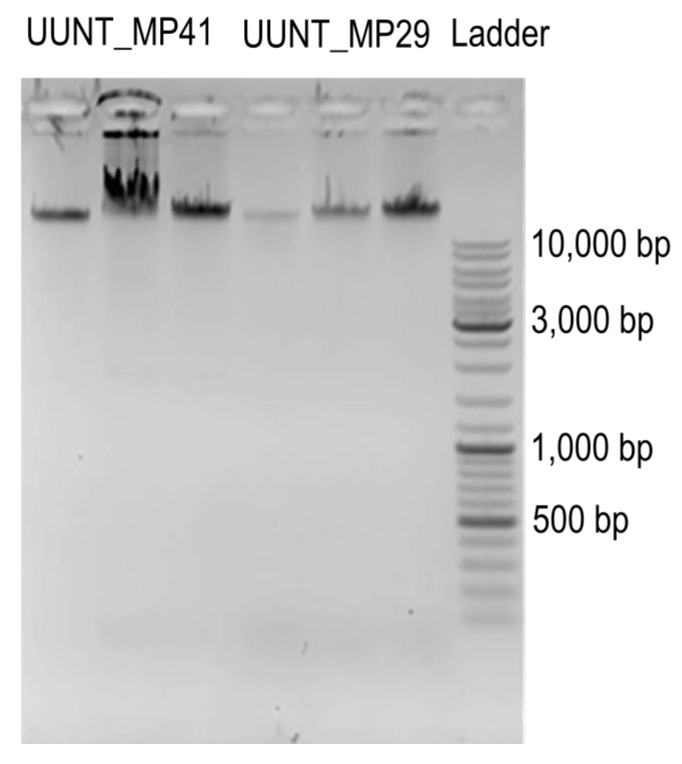
Gel electrophoresis of genomic DNA isolated from strains *H. alvei* UUNT_MP41 and *H. paralvei* UUNT_MP29.

Thirty-nine bacterial strains were isolated from the gut of healthy *C. carpio* and were identified using 16S rRNA gene amplification and sequencing. Bacterial strains identified at the species level using standard/universal 16S rRNA gene primers (27F-AGAGTTTGATCCTGGCTCAG and 1492R-ACGGYTACCTTGTTACGACTT) are presented in Table 1. From 39 strains, 6 genera were recognized and presented in the GIT of *C. carpio*. According to the morphological characteristics of the strains, such as shape, size, and colors, two strains of *Hafnia* species, *H. alvei* UUNT_MP41 and *H. paralvei* UUNT_MP29, are selected for further studies. These strains have been selected for further study/research due to their classification as possible next-generation probiotics (NGP), which makes them particularly appealing now. We extracted genomic DNA from these strains in three independent replicates (Figure 1) and performed 16S rRNA gene sequencing, followed by whole-genome next-generation sequencing (NGS).

### 2.2. Genome Analysis of H. paralvei UUNT_MP29 and H. alvei UUNT_MP41 Strains

**Figure 2:**
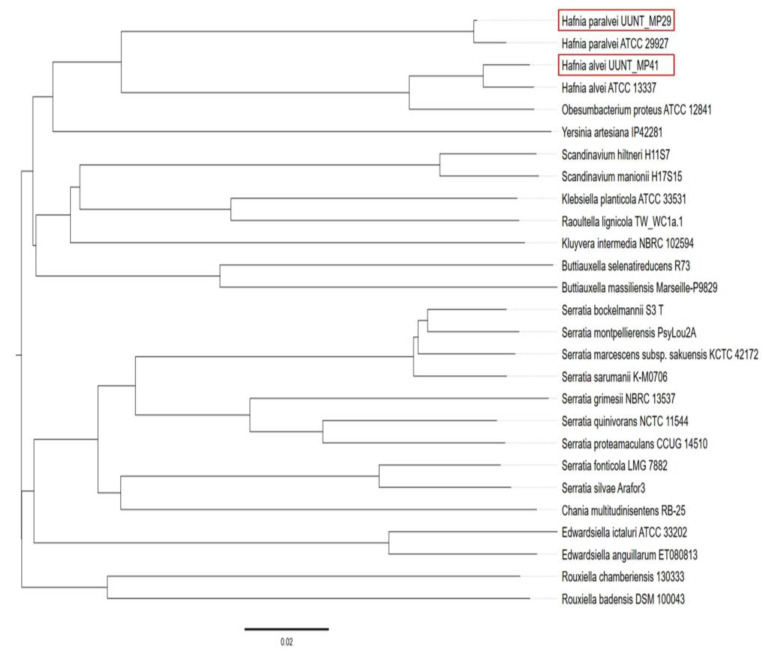
Intergeneric Phylogenetic Tree. The closest related species from the TYGS database were selected for the construction [21]. The phylogenetic trees were inferred with FastME 2.1.6.1 [22] from GBDP distances calculated from genome sequences. The branch lengths are scaled using the GBDP distance formula d5 [23]. The tree was rooted at the midpoint. New *Hafnia* species are marked in red squares.

To analyze the genomes for the search for potentially interesting genes, we performed NGS sequencing of both *Hafnia* strains. The assembled genomes have been deposited in the NCBI database [20] under BioProject numbers PRJNA1185451 for UUNT_MP41 and PRJNA1185452 for UUNT_MP29. Based on the Kraken analysis results (the results are shown in the Supplementary File Table S1, “Taxonomic Distribution” sheet), strain UUNT_MP29 is most closely related to the species *H. paralvei*, while strain UUNT_MP41 is most closely related to the species *H. alvei*. Likewise, the phylogenetic tree was constructed on a comparison of the complete genomes with the 10 closest strains from the TYGS database [21]. The results of this phylogenetic tree analysis also showed that strains isolated in this study cluster with the genus *Hafnia* (Figure 2).

**Figure 3:**
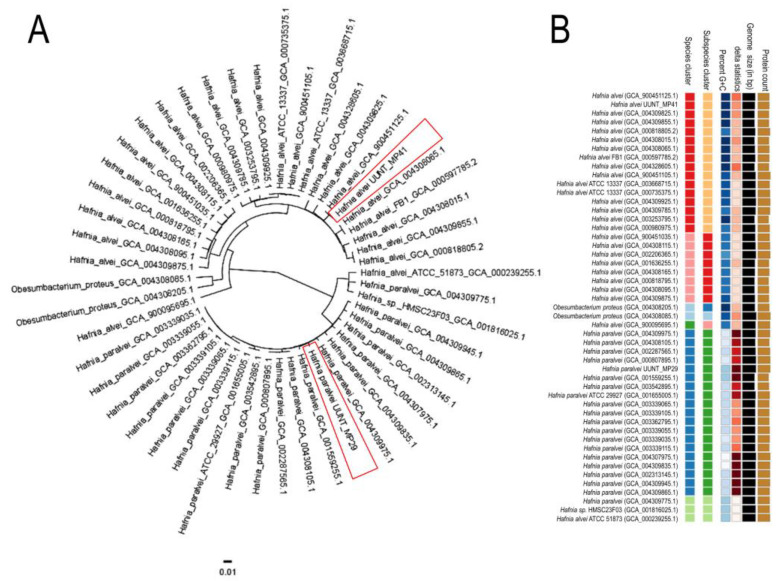
Panel (**A**)—Phylogenetic Tree of the *Hafnia* Genus. The list of genomes from the publication [24] was used to construct the phylogenetic tree. The phylogenetic trees were inferred with FastME 2.1.6.1 [22] from GBDP distances calculated from genome sequences. The branch lengths are scaled using the GBDP distance formula d5 [23]. The tree was rooted at the midpoint. Panel (**B**)—Characteristics of Hafnia Genus Members: Column 1—Affiliation to species clusters (dDDH species cluster [23,25,26]), Column 2—Affiliation to subspecies clusters (dDDH subspecies cluster [23,25,26]), Column 3—Genomic G+C content (lighter color—lower G+C content, darker color—higher G+C content percentage), Column 4—δ values (δ values guide the suitability of specific query genome sequences and the reliability of the phylogenetic outcome, with lighter shading indicating smaller δ values and more intense shading indicating higher δ values [26,27,28], Column 5—genome size in bp, Column 6—number of protein-coding open reading frames. New *Hafnia* species are marked in red squares.

For a more detailed phylogenetic analysis, we constructed an intra-genus tree with other representatives from the genus *Hafnia*, which we obtained from a comparative genomic analysis study of this genus [24]. The closest strain to UUNT_MP29 was the GenBank assembly GCA_001559255.1, and for UUNT_MP41, it was the GenBank assembly GCA_900451125.1 (Figure 3).

To characterize the antibiotic resistance of the studied strains, we used the CARD database (https://card.mcmaster.ca/analyze, accessed on 24 October 2024) [29]. The results are presented in the Supplementary File (Table S3). In the genome of *H. paralvei* UUNT_MP29, two genes with a “Perfect” rating according to CARD’s RGI Criteria [30]—blaACC-1 and blaTEM-116—were found. Both genes encode beta-lactamase enzymes [31,32] and may confer resistance to antibiotics such as cefepime, ceftazidime, piperacillin, aztreonam, cefotaxime, cefotetan, flomoxef, moxalactam, cefpirome, and ampicillin. Additionally, 10 genes with a “Strict” rating in CARD were identified, which may confer resistance to erythromycin, gentamicin, ciprofloxacin, spectinomycin, streptomycin, tobramycin, ceftazidime, ertapenem, piperacillin, azithromycin, imipenem, polymyxin B (and its variants), norfloxacin, ticarcillin, colistin, tetracycline, cefepime, ceftriaxone, rifampin, triclosan, benzalkonium chloride, chlorhexidine, trimethoprim, chloramphenicol, cloxacillin, oxacillin, ampicillin, cefaclor, cefotaxime, cefditoren, cefdinir, and pulvomycin.

In the genome of *H. alvei* UUNT_MP41, one gene with a “Perfect” rating according to CARD—QnrD1, which encodes a quinolone resistance pentapeptide repeat protein and is associated with resistance to fluoroquinolones [33]—was found. Additionally, 11 genes with a “Strict” rating in CARD were identified, which may confer resistance to a similar spectrum of antibiotics as mentioned for strain *H. paralvei* UUNT_MP29.

These data partially confirm the results obtained in in vivo experiments on the antibiotic resistance of the studied strains (shown below): both strains are resistant to certain penams (ampicillin, amoxicillin, amoxicillin-clavulanic acid, piperacillin-tazobactam) and cephalosporins (ceftazidime, cefotaxime). Although homologs of resistance genes to carbapenems, fluoroquinolones, and aminoglycosides were found in the genomes of both strains, these strains were sensitive to compounds from these groups (imipenem, ciprofloxacin, enrofloxacin, gentamicin, neomycin, and tobramycin) under the tested conditions. This could be due to insufficient gene expression or other factors.

We also investigated the presence of virulence factor genes in the genomes using the VFDB database [34]. In both genomes, genes from the VF categories adherence, antiphagocytosis, invasion, iron uptake, secretion system, anaerobic respiration, autotransporter, biofilm formation, efflux pump, fimbrial adherence determinants, immune evasion, and nutritional factor were identified. UUNT_MP41 also contained a gene from the VF category lipid and fatty acid metabolism and a gene encoding O-antigen (the results are shown in the Supplementary Materials, Table S4).

The pathogenicity and toxicity of the studied strains will be further investigated in in vivo experiments.

### 2.3. Antibiotic Susceptibility of H. alvei UUNT_MP41 and H. paralvei UUNT_29

**Figure 4:**
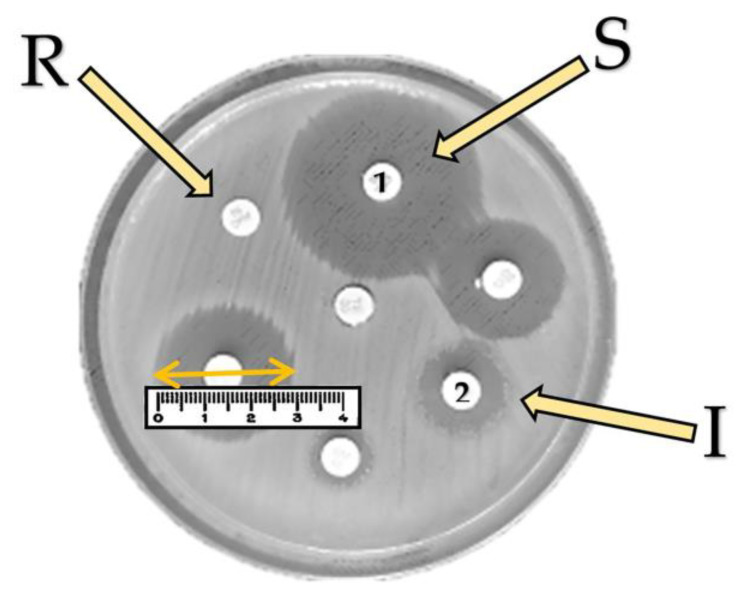
Disk diffusion test results at 37 °C: Sensitive (S)—expected success of therapy with the given antibiotic; Intermediate (I)—sensitive at increased exposure; Resistant (R)—high probability of therapeutic failure. 1—Ciprofloxacin 5 μg/disc; 2—Tetracycline 30 μg/disc; 3—Amoxicillin 25 μg/disc.

To investigate the strains further, we determined their resistance spectrum to 15 antibiotics in in vivo experiments (Figure 4). The obtained data were similar to those found in a review of other *Hafnia* species [24]. The final classification of the strains based on resistance/sensitivity is provided in Table 2. We classified the strains into three groups: R—resistant, S—sensitive, and I—intermediate result. The classification was assigned according to the standards of the Clinical and Laboratory Standards Institute for Enterobacteriales [35].

**Figure 5:**
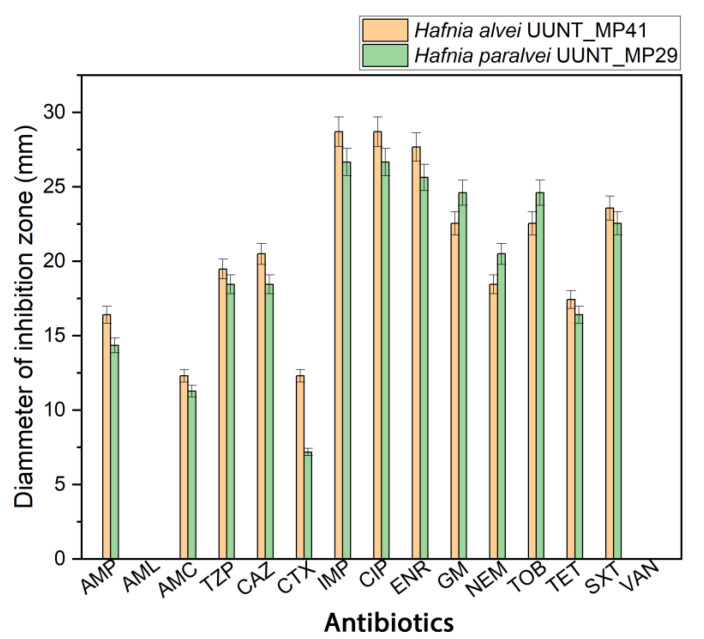
Disc diffusion method for assessing antibiotic susceptibility of *H. alvei* UUNT_MP41 and *H. paralvei* UUNT_MP29. AMP—Ampicillin; AML—Amoxicillin; AMC—Amoxicillin-clavulanic acid; TZP—Piperacillin-Tazobactam; CAZ—Cephalosporins; CTX—Ceftazidime; IMP—Imipenem; CIP—Ciprofloxacin; ENR—Enrofloxacin; GM—Gentamicin; NEM—Neomycin; TOB—Tobramycin; TET—Tetracycline; SXT—Sulfamethoxazole-Trimethoprim; VAN—Vancomycin.

Based on the results of the trials, it was determined that both strains exhibited the maximum level of sensitivity to imipenem, ciprofloxacin, and enrofloxacin. In the group of carbapenems, imipenem demonstrated that both strains of *H. alvei* UUNT_MP41 (28.70 mm) and *H. paralvei* UUNT_29 (26.65 mm) were sensitive to the antibiotic. The values that were shown here were the identical values for ciprofloxacin from the fluoroquinolone group. These values represent the maximum sensitivity of both strains to these two antibiotics. Compared to ciprofloxacin and imipenem, the inhibition zones on enrofloxacin showed that these strains had a lower susceptibility to the antibiotic. The resistance zone for *H. alvei* UUNT_MP41 was tested to be 27.67 mm, whereas the resistance zone for *H. paralvei* UUNT_29 was measured to be 25.62 mm. On the other hand, *H. alvei* UUNT_MP41 was found to be sensitive to both ampicillin and ceftazidime, while *H. paralvei* UUNT_29 was shown to have an intermediate sensitivity to both antibiotics. Despite the existence of the β-lactamase gene in the genome of both strains, which is the enzyme responsible for resistance to ceftazidime, both strains of bacteria exhibited sensitivity to this antibiotic. They did not exhibit any evidence of resistance. Additionally, it was shown that these strains were resistant to five different medications: amoxicillin, amoxicillin-clavulanic acid, piperacillin-tazobactam, cefotaxime, and vancomycin (Figure 5). These antibiotics were used to treat the infection. The absence of inhibitory zones on amoxicillin and vancomycin is evidence that these strains may be able to demonstrate probiotic activity (NGP) [8]. This is because the *H. alvei* strain is considered a member of the new generation of probiotics (NGP) [8], developed throughout the past few years.

### 2.4. Antimicrobial Activity of H. alvei UUNT_MP41 and H. paralvei UUNT_29

Potential probiotic organisms are assessed on antimicrobial activity and susceptibility of the antibiotics, which are the main characteristics of the probiotics. In this study, *H. alvei* UUNT_MP41 and *H. paralvei* UUNT_29 have been assessed on eight different pathogens using the disk diffusion method. Eight pathogen strains, *Enterococcus faecalis* ATCC 29212, *Staphylococcus aureus* subsp. *aureus* strain ATCC 6538, *Pseudomonas aeruginosa* ATCC 27853, *Streptococcus pneumoniae* ATCC 6301, *Bacillus subtilis* ATCC 6633, *Shigella flexneri* ATCC 12022, *Klebsiella pneumoniae* subsp. *pneumonia* ATCC 13883 and *Escherichia coli* ATCC 25922 were used to determine the antimicrobial properties of the *H. alvei* UUNT_MP41 *and Hafnia paralvei* UUNT_29 strains. The fragile inhibitory zones were obtained after the treatment with these strains against pathogens. *Pseudomonas aeruginosa* ATCC27853 and *Staphylococcus aureus* ATCC 6538 were the only two pathogenic strains that the strains *H. alvei* UUNT_MP41 and *H. paralvei* UUNT_29 were able to suppress, albeit in a very mild manner.

### 2.5. Search for a Potential Candidate Gene for Plastic Degradation

Additionally, we have tried to determine the gene accountable for the biodegradation of polystyrene in both strains. We selected protein sequences that are known to be involved in the decomposition of plastic. We did a BLAST search (blastp) [36] of annotated proteins in *Hafnia* genomes against protein sequences from this database. This search was based on the Plastics Microbial Biodegradation Database (PMBD) [37]. The selection of proteins from the database was limited to those that were confirmed and whose sequences were accessible through the UniProt database [38].

Considering the findings, we chose the five candidate proteins that showed the most promise and presented them in the (Supplementary Materials Table S5). It was discovered that homologs of arylesterase *PpEst* (https://www.uniprot.org/uniprot/A0A145Z9W5, accessed on 5 November 2024) were present in both the *H. paralvei* UUNT_MP29 and the *H. alvei* UUNT_MP41 genomes. This protein obtained the highest BLAST score compared to other proteins in our in-house database created from PMBD. The proteins from *H. paralvei* UUNT_MP29 (282940_A29_00559) and *H. alvei* UUNT_MP41 (282941_A41_00661) were similar. Furthermore, the *PpEst* from *Pseudomonas pseudoalcaligenes* shared more than 56% of its identity with the *H. paralvei* UUNT_MP29 protein. It is well known that *PpEst* can hydrolyze the co-aromatic-aliphatic polyester poly (1,4-butylene adipate-co-terephthalate) [39].

### 2.6. Investigation of the Presence of the Heat Shock Protein Gene ClpB Involved in Conferring Probiotic Properties to Bacteria

The initial step in the process of determining whether or not the strains *H. alvei* UUNT_MP41 and *H. paralvei* UUNT29 possess the gene that codes for the ATP-dependent chaperone *ClpB* (heat shock protein) that has the α-MSH epitope was to determine whether or not these strains are potential candidates for the use of probiotics in the control of body weight. The analysis showed that both strains possess the gene for the *ClpB* protein, which at critical positions has the corresponding conservative amino acids (E539, R543, W544, G546, P548, and V549) responsible for antigen-mimetic of the anorexigenic α-melanocyte stimulating hormone (α-MSH) and the body weight control activity [8,40,41].

## 3. Discussion

Probiotics are “live microorganisms that, when administered in adequate amounts, confer a health benefit on the host” [42]. Probiotics are beneficial to the health of the host. Several different genera are utilized as probiotics, such as *Lactobacillus*, *Bifidobacterium*, *Bacillus*, *Pediococcus*, and various yeasts [43]. Recent developments in the study of the gut microbiome, which are still ongoing, have shed light on the role of the gut microbiota as a source of potentially beneficial probiotic bacteria that has not been thoroughly investigated. These days, there is a growing interest in the next generation of probiotics, which include bacteria such as *Eubacterium hallii*, *Faecalibacterium prausnitzii*, *Roseburia spp*., *Akkermansia muciniphila*, and *Bacteroides fragilis*, as potential biotherapeutics that can change the microbiome of the gut and influence the development of a variety of diseases [44].

A targeted strategy and screening for bacteria that generate ClpB, a protein that mimics alpha-MSH and activates satiety signals, led to the development of *H. alvei*, the first precision probiotic created according to this methodology. As a result of its method of action and established efficacy, it is a probiotic of the next generation that is revolutionary, inventive, and uniquely positioned for weight management [8,45,46]. This study intended to extract and describe cultivable strains from the gastrointestinal tract of common carp that can potentially be used as probiotics for both human and veterinary purposes. Taking into consideration the fact that the selection of strains was carried out in this study to maintain the temperature at 37 °C, it is reasonable to anticipate that the representation of species will be different from that of studies in which the analysis of the complete microbiome was carried out [47,48,49,50]. *Citrobacter* species are represented in a way consistent with other researchers’ findings, although *Serratia* and *Kocuria* species are the most prevalent in this investigation. Additionally, the separation of *H. alvei* and *H. paralvei* indicates the microbiota of fish kept in farm environments [47]. Given that two autochthonous strains of the genus *Hafnia* were isolated in this study (*H. alvei* UUNT_MP41 and *H. paralvei* UUNT29), both of which have the potential to inherit probiotic properties, a more in-depth analysis of these strains was carried out. This analysis included the following: antimicrobial activity against common pathogens, antibiotic resistance, whole genome sequencing, and their bioinformatic analysis. After doing genomic analysis on these two strains, it was discovered that the separated strains have characteristics that place them in the category of common strains of the species in question [24].

The commensal bacteria *H. alvei* and *H. paralvei* are parallel and essential components of the microbiota in the digestive tract. Yet, certain strains of these bacteria can potentially cause disease [51]. To maintain control over bacteria utilized in biotechnology or as probiotics, it is essential to understand their susceptibility to antibiotics and the likelihood that they will transfer genes that confer resistance to other bacteria. A genome study of *H. alvei* UUNT_MP41 and *H. paralvei* UUNT29 strains was carried out to determine whether or not antibiotic-resistance genes were present. Additionally, antibiogram tests were carried out and analyzed. After doing an investigation using bioinformatics, it was discovered that the genomes of *H. alvei* UUNT_MP41 and *H. paralvei* UUNT29 strains contained a significant number of genes that were responsible for resistance to a variety of antibiotics. These drugs included beta-lactam-type antibiotics, aminoglycoside antibiotics, polymixins, and others. These findings are in keeping with the findings of other groups, and they are regarded as intrinsic because they are located on chromosomes, where there is a limited chance of transfer to different/other bacterial species. Furthermore, our research demonstrated that *H. paralvei* UUNT29 has a higher level of resistance to antibiotics than *H. alvei* UUNT41, which has already been shown and demonstrated [47,52].

According to the findings of the antibiogram test, *Hafnia* strains exhibit susceptibility to imipenem, ciprofloxacin, and enrofloxacin. On the other hand, they are resistant to amoxicillin, amoxicillin-clavulanic acid, piperacillin-tazobactam, cefotaxime, and vancomycin, indicating they are appropriate for further application. In light of the findings of this investigation, it is possible to conclude that, despite the limitations of the selection conditions, a significant number of distinct strains belonging to six different genera and nine different species were isolated. Two species, *H. alvei* and *H. paralvei*, have been isolated as potential probiotics. These strains can be utilized to manage body weight and the degradation of plastics. This is because they possess genetic characteristics, such as the ClpB heat shock protein gene and many enzymes associated with microplastics’ degradation. In particular, the presence of a gene that shares a high percentage of identity with PpEst from *Pseudomonas pseudoalcaligenes*, will be the focus of further investigation. The first findings of feeding mice with strains of *H. alvei* UUNT_MP41 and *H. paralvei* UUNT29 demonstrated that these strains do not adversely impact the development and health of the treated mice. This result provides a solid foundation for further research.

## 4. Materials and Methods

### 4.1. Isolation of Autochthonous Species from the Gastrointestinal Tract of C. carpio

**Scheme 1:**
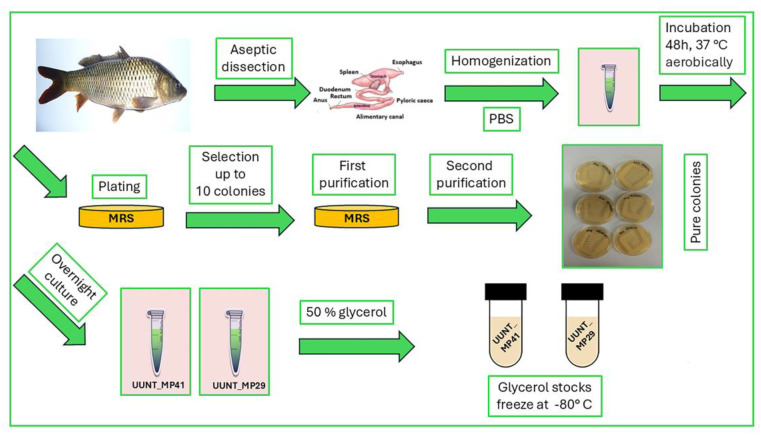
Isolation of *Hafnia* species from *C. carpio*.

The fish samples were collected from the fishpond “DTD” in Backi Jarak, Serbia, to isolate and identify indigenous bacterial strains from the gastrointestinal tract (GIT) microbiota of *C. carpio*. The samples were brought to the laboratory alive and sacrificed. The fish samples weighed an average between 1700 and 1850 g. The longitude sectioning of the samples was performed under aseptic conditions, and the abdominal surfaces of all samples were meticulously cleansed with 70% ethanol. The specimens are washed three times and dissected aseptically to remove the intestines. The samples’ intestines were longitudinally incised and extensively rinsed with sterile phosphate buffer saline (PBS) to eliminate feed debris, dirt, and other contaminants. The weight of the intestines was measured, and then samples were macerated using sterile scissors. The homogenization was conducted in sterile PBS (1:10 wet/vol) using a vortex mixer. Homogenized samples were serially diluted in PBS and plated aseptically using the pour plate technique on Man Rogosa Sharpe (MRS) agar plates. The inoculated MRS plates are incubated at 37 °C for 48 h under aerobic conditions. Colonies exhibiting distinct morphological variations, including differences in size, color, and form, were chosen and isolated/repurified by streaking two consecutive passages onto MRS plates. The pure strains were grown overnight (16 h) in MRS broth, and 50% glycerol (Sigma-Aldrich, St. Louis, MO, USA) was added to make glycerol stocks. The strains were stored at −80 °C, until use in further experiments [53,54]. The isolation procedure is depicted in Scheme 1.

### 4.2. Species Identification by 16S rRNA Gene Sequencing

The identification of isolated strains was conducted using Sanger sequencing of the 16S rRNA gene amplified using standard primers (27F-AGAGTTTGATCCTGGCTCAG, and 1492R-ACGGYTACCTTGTTACGACTT). DNA fragment amplification was performed using thermal cycler Mastercycler Pro (Eppendorf) in the following manner: initial denaturation at 94 °C for 5 min, followed by 30 cycles consisting of denaturation at 94 °C for 1 min, annealing at 55 °C for 30 s, and polymerization at 72 °C for 1 min, and a final extension at 72 °C for 7 min. Confirmation of amplification of appropriate DNA fragments was performed using 1% agarose gel electrophoresis. Five µL of the amplified PCR fragments/reactions were loaded onto 1% agarose gel in the TAE buffer (40 mM Tris-acetate, 1.0 mM EDTA, pH 8.2). Agarose gel was stained with ethidium bromide (500 ng/mL) and visualized under UV light (EZEE gelONE, Cleaver Scientific, Rugby, UK). DNA fragments were sized with gene ruler (GR), GeneRuler DNA Ladder Mix, from 100 to 10,000 bp. Sanger sequencing was performed on an Applied Biosystems 3500 Genetic Analyzer. The PCR product clean-up was performed using ExoSAP-IT PCR Product Cleanup Reagent following the manufacturer’s protocol. The sequencing reaction setup was performed using the BigDye Terminator v3.1 Cycle Sequencing Kit according to the manufacturer’s protocol. Centri-Sep columns (Applied Biosystems, Waltham, MA, USA) were used for reaction clean-up before loading onto the device. Before loading onto capillary electrophoresis, 15 μL of formamide was added to 2 μL of the cleaned sequencing product. The Basic Local Alignment Search Tool [36] algorithm was used to determine the most related sequences in the NCBI nucleotide sequence database [20].

### 4.3. Genomic DNA Isolation

DNA was extracted using the GeneJet Genomic DNA Purification Kit (Thermo Scientific, Waltham, MA, USA) following the manufacturer’s protocol and instructions. The purity and concentration of the extracted DNA were assessed using a spectrophotometer Nanodrop 2000c (Thermo Scientific) with visualization of the final samples on agarose gel electrophoresis.

### 4.4. NGS Sequencing and Bioinformatics Analysis

The extracted genomic DNA was sequenced using the Illumina Short Reads platform (2 × 250 bp) with 30X target coverage. The reads were trimmed using Trimmomatic (v. 0.39) [55], and the quality was assessed using in-house scripts combined with the following software: Samtools (v. 1.19.2) [56], BedTools (v. 2.28.0) [57], and bwa-mem (v. 0.7.15-r1140) [58]. A table showing the characteristics of the trimmed reads used in the analysis is available in the Supplementary File (Table S1, “Trimmed Reads” sheet). Genome assembly was performed using SPAdes (v. 4.0.0) [59]. The final genome assembly statistics are presented in the Supplementary File (Table S1, “Assemblies” sheet). Paired-end and single-end reads obtained from Illumina sequencing were used as input data. The final genome assembly statistics are presented in the Supplementary File (Table S1, “Assemblies” sheet). The assembled genomes have been deposited in the GenBank repository under the following accession numbers (to be inserted). The annotation of the assembled genomes was carried out using Prokka (v. 1.14.6) [60]. A list of all protein-coding genes is provided in the Supplementary File (Table S2). Taxonomic distribution analysis was performed using Kraken (v. 2.1.2) [61].

The phylogenetic tree was constructed using the bioinformatics platform Type (Strain) Genome Server (TYGS) [21,26]. For intergeneric phylogenetic tree construction, TYGS’s sister database, the “List of Prokaryotic Names with Standing in Nomenclature” [21], was used. For intrageneric phylogenetic tree construction, a list of genomes from the publication [24] was applied. Identification of genomes of the closest type strains was performed using the MASH algorithm for rapid intergenomic similarity approximation [62], utilizing all type strain genomes available in the TYGS database [21]. Consequently, the type strains with the smallest MASH distances were selected for each genome. For phylogenomic inference, all pairwise comparisons between genome sets were performed using GBDP with precise intergenomic distances calculated via the “trimming” algorithm and distance formula d5 [23]. For each case, 100 distance replicates were computed. Digital DDH values and confidence intervals were calculated using the recommended settings in GGDC 4.0 [21,23]. The obtained intergenomic distances were used to generate a balanced minimum evolution tree with branch support through FASTME 2.1.6.1, including SPR post-processing [22]. Branch support was derived from 100 pseudo-bootstrap replicates each. The trees were rooted at the midpoint [63] and visualized using PhyD3 [64] and FigTree (http://tree.bio.ed.ac.uk/). Species clustering based on type was performed using a 70% dDDH radius around each of the type strains, as previously described [26]. Subspecies clustering was conducted with a threshold of 79% dDDH, as outlined in earlier studies [25]. The assembled genomes were analyzed using the CARD database [29] to identify antibiotic resistance genes. The assembled genomes were also analyzed using the VFDB database for the presence of virulence factors [34]. The results are presented in the Supplementary File (Table S4).

### 4.5. Pathogen Strains, Media, Culture Conditions, and Antimicrobial Activity Assay

Pathogen bacterial cultures were obtained from the collection of The Institute of Virology, Vaccines, and Sera “Torlak”. Table 3 presents selected pathogen strains for determining the antimicrobial activity of autochthonous strains isolated from *C. carpio*. *Enterococcus faecalis* ATCC 29212 was grown in an M17 medium supplemented with 0.5% (*w*/*v*) glucose (GM17) at 30 °C. *Staphylococcus aureus* subsp. *aureus* strain ATCC 6538, *Pseudomonas aeruginosa* ATCC 27853, *Streptococcus pneumoniae* ATCC 6301, *Bacillus subtilis* ATCC 6633, *Shigella flexneri* ATCC 12022, *Klebsiella pneumoniae* subsp. *pneumoniae* ATCC 13883 and *Escherichia coli* ATCC 25922 were grown aerobically in Luria-Bertani medium (LB) at 37 °C. A 0.7% agar nutrient medium was used for creating bacterial lawns, and a 2% agar nutrient medium was used for simple bacterial cultivation. The agar disk diffusion method, as described by Popovic et al. [65], with specific alterations, was employed to assess the antimicrobial efficacy of indigenous strains obtained from *C. carpio*. Briefly, cell-free supernatants from overnight cultures of autochthonous strains (~108 CFU/mL) in MRS broth were collected by centrifugation at 2800 g at 4 °C for the next 10 min, and 10 µL of the overnight culture or 10 µL of cells diluted in 1X TE buffer (10 mM Tris, 1.0 mM EDTA, pH 7.5), that was filter sterilized (0.22 mm pore size filter). Fifty μL of cell-free supernatant was spotted on 6 mm diameter filter paper disks and placed over the surface of 0.7% melted agar nutrient medium (Torlak, Serbia) that was sterilized (Torlak, Serbia), seeded with 100 μL of each pathogen species containing 105 CFU/mL. The plates were incubated at 37 °C, except *Enterococcus faecalis* ATCC 29212, at 30 °C for 24 h. The radius of the inhibition zone was measured at the net of the disk’s radius. The result obtained from this experiment was documented as the average of three distinct tests. Positive control broad spectrum multi-antimicrobials producer *Brevibacillus laterosporus* BGSP11 was used [66].

### 4.6. Antibiotic Susceptibility of H. alvei UUNT_MP41 and H. paralvei UUNT_MP29 Strains

*H. alvei* UUNT_MP41 and *H. paralvei* UUNT29 were assessed with the disk diffusion method (Kirby–Bauer test) on the reference medium Mueller–Hinton agar (MHA) to determine the antibiotic susceptibility of these autochthonous strains. Overnight cultures of *H. alvei* UUNT_MP41 and *H. paralvei* UUNT29 were aerobically inoculated into Mueller–Hinton broth at 37 °C. 100 μL of diluted culture (~10^8^ CFU/mL) were spread onto MHA plates of each strain, and the antibiotic discs were applied onto the surface of the plates. The following antibiotic discs were dispensed on the medium: penicillins, cephalosporins, carbapenems, fluoroquinolones, aminoglycosides, as well as tetracycline, sulfamethoxazole-trimethoprim, and vancomycin used as negative control. The concentrations of indicator antibiotics and antibiotics are presented in Table 4. Incubation of the plates was conducted at 37 °C for 48 h, followed by an analysis of the inhibition zone sizes. All the results of the measured inhibition zones around the antibiotic discs are presented in millimeters.

## 5. Conclusions

This study aimed to isolate and describe cultivable strains from the GIT of common carp that can potentially be used as probiotics for both human and veterinary purposes. Despite the limitations of the selection conditions, a significant number of distinct strains belonging to six different genera and nine different species were isolated. Two autochthonous strains of the genus *Hafnia* were isolated, which could inherit probiotic properties. Genome analysis and antibiogram tests were conducted on these strains to determine their antibiotic resistance. The results showed that *H. alvei* UUNT_MP41 and *H. paralvei* UUNT_MP29, have significant resistance to various antibiotics, including imipenem, ciprofloxacin, and enrofloxacin, but are resistant to amoxicillin, amoxicillin-clavulanic acid, piperacillin-tazobactam, cefotaxime, and vancomycin. According to their characteristics, these two strains, *H. alvei* UUNT_MP41 and *H. paralvei* UUNT_MP29, could be considered candidates for a potential new generation of probiotics that can be used for body weight control and plastic degradation. Further investigation will focus on a complete analysis of the safe use of these strains and on a gene that shares a high percentage of identity with PpEst from *Pseudomonas pseudoalcaligenes* for the biodegradation of microplastics.

## Figures and Tables

**Table 1:** Identified species isolated from GIT of C. carpio using 16S rRNA sequencing.

Identified Genus	Identified Species	No of Strains
*Citrobacter*	*Citrobacter freundii*	13
*Serratia*	*Serratia liquefaciens*	10
	*Serratia plymuthica*	1
	*Serratia marcescens*	1
*Bacillus*	*Bacillus pumilus*	3
*Hafnia*	*H. alvei*	1
	*H. paralvei*	1
*Enterococcus*	*Enterococcus pseudoavium*	1
*Kocuria*	*Kocuria rhizophila*	8

**Table 2:** Results of Disk Diffusion Test.

Antibiotics	Microorganism (Bacteria)			
	*H. alvei*		*H. paralvei*	
	Zone Diameter, mm	Classification	Zone Diameter, mm	Classification
PENICILINS				
Ampicilin	16	I	14	I
Amoxicilin	0	R	0	R
Amoxicilin-clavulanic acid	12	R	11	R
Piperacilin-Tazobactam	19	R	18	R
CEPHALOSPORINS				
Ceftazidime	20	I	18	I
Cefotaksime	12	R	7	R
CARBAPENEMS				
Imipenem	28	S	26	S
FLUOROQUINOLONES				
Ciprofloxacin	28	S	26	S
Enrofloxacin	27	S	25	S
AMINOGLYCOSIDES				
Gentamicin	22	S	24	S
Neomycin	18	S	20	S
Tobramycin	22	S	24	S
OTHER ANTIBIOTICS				
Tetracycline	17	I	16	I
Sulfametoxazol-Trimetoprim	23	S	22	S

**Table 3:** Pathogen strains, media, and culture conditions for determination of antimicrobial activity.

Pathogen Strain	Medium	Temperature, °C
*Enterococcus faecalis* ATCC 29212	M17	30
*Staphylococcus aureus subsp. aureus strain* ATCC 6538	LB	37
*Pseudomonas aeruginosa* ATCC 27853	LB	37
*Streptococcus pneumoniae* ATCC 6301	LB	37
*Bacillus subtilis* ATCC 6633	LB	37
*Shigella flexneri* ATCC 12022	LB	37
*Klebsiella pneumoniae* subsp. *pneumoniae* ATCC 13883	LB	37
*Escherichia coli* ATCC 25922	LB	37

**Table 4:** Concentration of antibiotics discs for disk diffusion method and referent values of Order Enterobacteriales (Family Enterobacteriaceae) ^1^.

Antibiotic	Referent Values of Disk Diffusion Test Inhibition Zones of Order Enterobacteriales (Family Enterobacteriaceae) ^1^			
	Concentration, μg per disc	R	I	S
Ampicillin	10	≤13	14–16	≥17
Amoxicillin	25	≤13	14–17	≥18
Amoxicillin-clavulanic acid	20/10	≤13	14–17	≥18
Piperacillin-Tazobactam	30/6	≤20	21–24	≥25
Ceftazidime	30	≤17	18–20	≥21
Cefotaxime	5	≤22	23–25	≥26
Imipenem	10	≤19	20–22	≥23
Ciprofloxacin	5	≤21	22–25	≥26
Enrofloxacin	30/6	≤16	17–21	≥22
Gentamicin	5	≤14	15–17	≥18
Neomycin	10	≤12	13–16	≥17
Tobramycin	10	≤12	13–16	≥17
Tetracycline	30	≤11	12–14	≥15
Sulfamethoxazole-Trimethoprim	23.75/1.25	≤10	11–15	≥16
Vancomycin	30	≤14	15–16	≥17

^1^ Concentration of antibiotic discs according to Performance standards for antimicrobial susceptibility testing, 34th edition [35].

## Data Availability

The data is contained within the article.

## Supplementary Materials

The following supporting information can be downloaded at: https://www.mdpi.com/article/10.3390/ijms26031119/s1.
